# Non‐lethal sampling does not misrepresent trophic level or dietary sources for *Sagmariasus verreauxi* (eastern rock lobster)

**DOI:** 10.1002/rcm.9435

**Published:** 2022-12-18

**Authors:** Jeremy Karl Day, Nathan Aaron Knott, Daniel Swadling, David Ayre, Megan Huggett, Troy Gaston

**Affiliations:** ^1^ School of Environmental and Life Sciences University of Newcastle Ourimbah New South Wales Australia; ^2^ NSW Department of Primary Industries Fisheries Research Huskisson New South Wales Australia; ^3^ School of Earth, Atmospheric and Life Sciences University of Wollongong Wollongong New South Wales Australia

## Abstract

**Rationale:**

Isotope analysis can be used to investigate the diets of predators based on assimilation of nitrogen and carbon isotopes from prey. Recent work has shown that tissues taken from legs, antennae or abdomen of lobsters can give different indications of diet, but this has never been evaluated for *Sagmariasus verreauxi* (eastern rock lobster). Work is now needed to prevent erroneous conclusions being drawn about lobster food webs, and undertaking this work could lead to developing non‐lethal sampling methodologies. Non‐lethal sampling for lobsters is valuable both ethically and for areas of conservation significance such as marine reserves.

**Method:**

We evaluated this by dissecting 76 lobsters and comparing δ^13^C and δ^15^N isotope values in antennae, leg and abdomen tissue from the same individuals ranging from 104 to 137 mm carapace length. Stable isotope values were determined using a Europa EA GSL elemental analyser coupled with Hydra 20–20 Isoprime IRMS.

**Results:**

We found the abdomen δ^13^C values to be lower than other tissues by 0.3 ± 0.2‰ for antennae tissue and 0.1 ± 0.2‰ δ^13^C for leg tissues, whereas for δ^15^N, no significant difference between tissues was observed. There was no significant effect of lobster size or sex, though we did observe interactions between month and tissue type, indicating that differences may be seasonal. Importantly, the detected range of isotopic variability between tissues is within the range of uncertainty used for discrimination factors in isotopic Bayesian modelling of 0‰–1.0‰ for δ^13^C and 3.0‰–4.0‰ for δ^15^N.

**Conclusions:**

We show that *S. verreauxi* can be sampled non‐lethally with mathematical corrections applied for δ^13^C, whereas any tissue is suitable for δ^15^N. Our results indicate that a walking leg is most favourable and would also be the least intrusive for the lobster. The application of non‐lethal sampling provides avenues for the contribution of citizen science to understanding lobster food webs and to undertake fieldwork in ecologically sensitive areas such as marine reserves.

## 1 INTRODUCTION

Globally, rock lobsters are considered to be important predators that can influence the community and trophic structure on temperate reefs. They are considered keystone species^1^, ^2^ as they can regulate sea urchin populations.^3^, ^4^, ^5^, ^6^, ^7^ However, the importance of the eastern rock lobster (*Sagmariasus verreauxi*) from the east coast of Australia as an urchin predator has recently been questioned.^8^

Stable isotope analysis enables the diet of animals to be quantitatively assessed by comparing the isotopes of carbon and nitrogen present in muscle and other tissues. Stable isotope analysis is possible because “stable” heavy isotopes of carbon (^13^C) and nitrogen (^15^N) do not undergo radioactive decay and are retained in tissues over their lighter isotopes (^12^C and ^14^N, respectively,^9^), and due to this, stable isotopes can be used to measure how energy is transferred through food webs. Given animals acquire the stable isotope composition of their muscle tissues from their diets, isotope analysis allows the structure of food chains and ecosystem linkages through trophic levels to be formally assessed.^11^, ^12^, ^13^ Isotopic changes are shown by delta notation “δ,” which is the convention for reporting ratios of ^13^C:^12^C (δ^13^C) and ^15^N:^14^N (δ^15^N) isotopes present in tissues, rather than an actual measurement for the isotopes themselves. Because isotope ratios derived from prey sources change through time with feeding, if a change in diet occurs, this will be matched by a change in the isotope profile of the consumer's muscle tissue.^12^, ^14^, ^15^ In ecological studies, significant changes in δ^13^C often indicate a change in the basal or immediate food source in predator diets,^12^, ^16^ whereas significant changes in δ^15^N are more frequently associated with a change in trophic level.^11^, ^12^ However, δ^13^C and δ^15^N are controlled by a myriad of biogeochemical factors, including how proteins are assimilated into tissues after feeding.^13^, ^17^ Consequently, different tissues can display different rates of isotopic turnover.^18^, ^19^ Indeed, previous studies which sampled a range of different tissue types to investigate diets have shown that tissue type can affect the conclusions drawn about predator diets.^12^, ^20^, ^21^, ^22^

We sampled the different white muscle tissues of *S. verreauxi* because when sampling crustaceans, internal tissues from the abdomen are usually removed and stored in the long term due to being protected within the animal’s carapace.^14^, ^18^, ^23^, ^24^, ^25^ This is in comparison to appendages like legs and antennae which are regularly broken off and regrown in the wild^26^, ^27^ and may present different isotope ratios.^28^, ^29^ Furthermore, sampling abdomen tissue necessitates the death of individuals.^12^, ^20^, ^21^, ^22^, ^23^, ^30^

Because some studies use non‐lethal tissues from lobsters on moral grounds,^13^, ^19^, ^31^ we wanted to determine whether non‐lethal sampling would be as informative for *S. verreauxi* as lethal sampling and whether potential isotope differences between tissues (appendage vs abdomen tissue) would be predictable. Importantly, as citizen involvement in science is becoming more common, there is potential for different tissue types to be donated (e.g., some lobsters may be supplied by divers without antennas), and therefore, whether one tissue type will be more valuable than another needs to be first evaluated to ensure comparable estimates of lobster diet. Here we aimed to determine whether non‐lethal tissue samples taken from the legs or antennae of *S. verreauxi* (eastern rock lobster) could be an alternative to lethal sampling of abdomen tissue. To evaluate the consistency of these tissue‐type estimates, we also assessed the influence of lobster size, sex and time of year on stable isotope ratios.

## 2 METHODS

### 2.1 Sample collection

**FIGURE 1:**
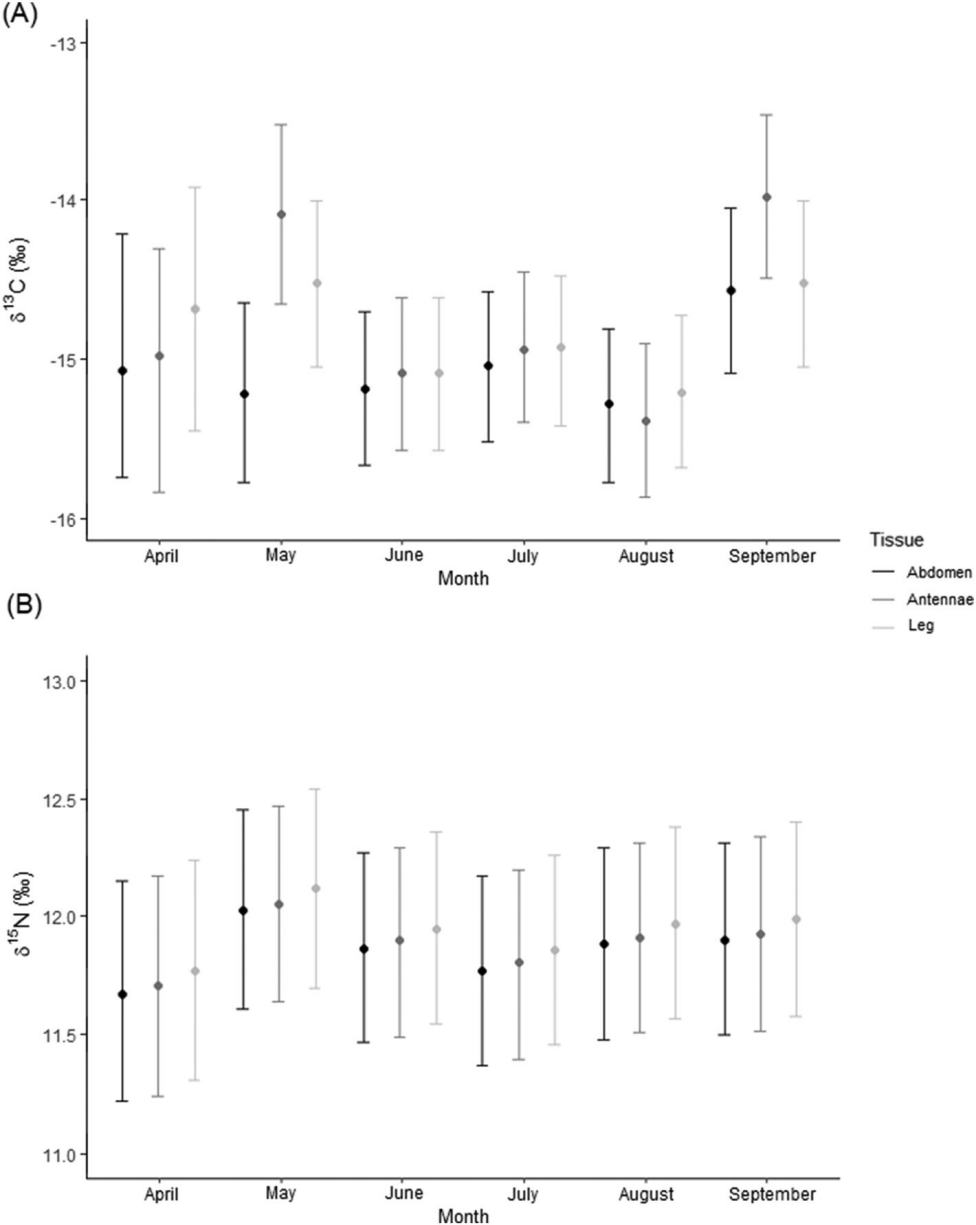
GLMM (generalised linear mixed model)‐predicted values for (A), δ^13^C and (B), δ^15^N showing isotopic differences by month collected. Tissue type (Abdomen, Antennae and Leg) is shown in colour, and the mean values for month and tissue type are shown plotted as points. Month collected and tissue type were within ±2 AICc units of the most parsimonious model for both isotopes

All lobster samples, including frozen carapaces donated by recreational divers, were hand‐collected on snorkel at Shellharbour, New South Wales, Australia, between July and September 2020 (Figure 1). The collected lobsters were euthanised in ice water before being stored at –18°C and thawed individually for dissection.^32^ We used abdomen muscle from inside the carapace of lobsters rather than the tail, which has been used in some studies (^19^; Table 1), because the donated lobster carapaces had their tails removed but abdomen tissue was present.

**TABLE 1:** Model candidates examining the effects of the different lobster tissues (tissue), lobster size (length), sex and month on values of (A) δ^13^C and (B) δ^15^N using the Akaike information criterion corrected for small sample sizes (AICc)

A						
Model	df	AICc	ΔAICc	Weight	*R* ^2^m	*R* ^2^c
^**13**^ **C ~ tissue + month + tissue × month + (1|lobster)**	**20**	**464.4**	**0.00**	**0.786**	**0.14**	**0.88**
^13^C ~ tissue + month + sex + tissue **×** month + (1|lobster)	21	467.1	2.75	0.199	0.15	0.88
^13^C ~ tissue + size + month + tissue **×** month + (1|lobster)	21	473.1	8.71	0.010	0.15	0.88
^13^C ~ tissue + month + sex + tissue **×** month + tissue **×** sex + (1|lobster)	21	475.7	11.36	0.003	0.15	0.88
^13^C ~ tissue + size + sex + month + tissue **×** month + (1|lobster)	23	476.0	11.65	0.002	0.15	0.88
^13^C ~ tissue + (1|lobster)	22	481.0	16.67	0.000	0.01	0.82
^13^C ~ tissue + sex + (1|lobster)	5	483.0	18.63	0.000	0.02	0.82
^13^C ~ tissue + size + sex + month + tissue **×** month + size **×** sex + (1|lobster)	6	483.8	19.43	0.000	0.15	0.88
^13^C ~ tissue + size + sex + month + tissue **×** month + sex **×** tissue + (1|lobster)	23	484.7	20.31	0.000	0.15	0.88
^13^C ~ tissue + month + (1|lobster)	24	485.7	21.32	0.000	0.10	0.83

B						
Model	df	AICc	ΔAICc	Weight	*R* ^2^m	*R* ^2^c
^**15**^ **N ~ (1|lobster) (NULL model)**	**3**	**421.5**	**0.00**	**0.320**	**0**	**0.21**
^**15**^ **N ~ month + (1|lobster)**	**8**	**421.9**	**0.35**	**0.269**	**0.11**	**0.23**
^**15**^ **N ~ tissue + (1|lobster)**	**5**	**422.7**	**1.23**	**0.173**	**0.03**	**0.25**
^**15**^ **N ~ tissue + month + (1|lobster)**	**10**	**423.3**	**1.77**	**0.132**	**0.14**	**0.27**
^15^N ~ sex + (1|lobster)	4	426.1	4.59	0.032	< 0.01	0.21
^15^N ~ month + sex + (1|lobster)	9	426.1	4.62	0.032	0.12	0.23
^15^N ~ sex + tissue + (1|lobster)	6	427.4	5.86	0.017	0.03	0.26
^15^N ~ size + (1|lobster)	11	427.6	6.08	0.015	< 0.01	0.21
^15^N ~ tissue + sex + tissue **×** sex + (1|lobster)	4	431.5	9.97	0.002	0.04	0.27
^15^N ~ month + sex + tissue + tissue **×** sex + (1|lobster)	8	431.8	10.25	0.002	0.15	0.28

*Notes*: This table shows the 10 most parsimonious GLMMs for each isotope, shown by the model with the fewest predictors with 2 AICc units of the model with the lowest AICc. The best model is shown first in bold font, and models within ±2 AICc units are also in bold font. Separate models were constructed for isotopes of δ^13^C and δ^15^N, and a lobster individual was included in all models as a random effect. Values for variation in the model contributed by fixed effects (*R* ^2^m) and random effects + fixed effects (*R* ^2^c) are included.

Abbreviation: GLMM, generalised linear mixed model.

### 2.2 Sample preparation

We recorded lobster sex, size and moult stage and excised ~1–2 g of white muscle tissue from the abdomen, antennae and leg of each lobster. Any lobsters which showed an upcoming or recent moult stage were not used as moulting may affect feeding.^6^, ^33^ We estimated the moult stage of lobsters by testing for softness at the limb margins of lobster carapaces and observing separation between layers of the exoskeleton.^8^ In all cases we used tissues from multiple appendages and took abdomen tissue from each individual.^19^

### 2.3 Stable isotope analysis

The samples were dried at 65°C for 24 h and then ground to a powder using a Retsch MM200 mixing mill. The ground samples were pre‐weighed (1–2 mg) into tin capsules, and stable isotope values were determined using a Europa EA GSL elemental analyser coupled to a Hydra 20‐20 automated Isoprime IRMS at the Stable Isotope Facility, Griffith University, Brisbane, Australia. Stable isotope ratios are conventionally expressed in delta notation (δ) relative to international reference materials for carbon (Vienna PeeDee Belemnite; VPDB) and nitrogen (atmospheric N_2_; AIR), where δ = (*R*_sample_/*R*_sample_ – 1) and *R*  = ^13^C/^12^C or ^15^N/^14^N (Fry et al 2006). The type of matrix used was simulated animal tissue composed of sucrose and ammonium sulphate, and this was calibrated against repeated measurements of an internal standard (NCSAT17A [working standard], IAEA‐N1 and IAEA‐N2 [^15^N standards] and IAEA‐CH‐6 [^13^C standard]), which produced standard deviations of ±0.1‰ for δ^13^C (*x̄* = −11.6 ± 0.1‰, n = 16) and δ^15^N (*x̄* = 0.2 ± 0.1‰, n = 16). Multiple point corrections were used to convert instrument data to internationally comparable values.

### 2.4 Statistical analysis

Relationships between δ^13^C and δ^15^N compared by tissue type, lobster size and sex and the month collected were examined using generalised linear mixed models (GLMM). Month was included as a predictor because it is unclear how lobster diets vary through time, and individual lobster IDs were included as a random effect in all models. Separate GLMMs were constructed for each response variable (δ^13^C and δ^15^N), and all possible model combinations were compared using Akaike's information criterion corrected for small sample sizes (AICc). AICc model selection is commonly used in ecological studies and was selected over traditional backward selection approaches because it allows all candidate models to be identified and compared. The “best” model was identified as having the lowest AICc value and highest weight.^34^ However, candidate models within ±2 AICc of the best model were also considered to have substantial support.^34^ When multiple candidate models occurred within ±2 AICc of the best model, the most parsimonious model (the model with the fewest predictors) was selected. To show *post hoc* sources of variation, we used the estimated marginal means method to compare the different tissue types by effect size shown with confidence intervals, as in Lei et al.^35^ We generated correction factors for isotope values of the different tissue types using the mathematical method described by Skinner et al.^36^ Finally, we used separate Pearson's correlations on raw data to test how well appendage tissue type (leg or antennae) could predict values for abdomen tissue.

All statistical analyses and plots were developed using the packages “lme4” (version 1.1.23, Foundation for Open Access Statistics, see https://cran.r-project.org/web/packages/lme4/index.html ^37^); for linear models, “MuMIn” (version 1.43.17, K. Barton, see https://cran.r-project.org/web/packages/MuMIn); for model comparisons, ggplot2 (version 3.3.0, see https://cran.r-project.org/web/packages/ggplot2/index.html ^38^); for plotting, dplyr (version 0.8.5, H. Wickham, R. Francois, L. Henry and K. Müller, see https://CRAN.R-project.org/package=dplyr); for sorting and cleaning data, emmeans (version 1.6.2‐1, V. Lenth, see https://CRAN.R-project.org/package=emmeans ^39^); and for comparing test statistics, R Studio (version 3.6.3, R Foundation for Statistical Computing, Vienna, Austria).

## 3 RESULTS

We analysed muscle tissue of the leg, antennae and abdomen from *S. verreauxi* of a range of carapace lengths (CL: 104–137 mm), including both males (n = 41) and females (n = 35). Seven lobster samples were collected by us, and 69 with all three tissue types (abdomen, antennae and leg) were donated.

### 3.1 δ^13^C in lobster tissues

AICc model comparisons showed the best model to include lobster tissue type, month collected and an interaction between tissue type and month collected (Table 1A). We found inconsistent variation between months, implying there was no discernible pattern in the way δ^13^C varied over the time periods tested (Figure 1A). However, δ^13^C increased in September compared to other months for all tissues, and antennae tissue also showed an additional increase in May. Overall, antennae tissues showed higher δ^13^C values than leg tissues, which were closer to abdomen tissues (Figures S1 and S2 [supporting information]). No other models were within ±2 AICc values of the most parsimonious model. Mean correction factors for δ^13^C in antennae and leg tissues were −0.3 ± 0.2‰ SE (standard error of the mean) δ^13^C and 0.1 ± 0.2‰ SE δ^13^C, respectively. Although we did not record any important effect of lobster size or sex, we did observe two trends: (a) δ^13^C decreased with increasing lobster size in all tissues (Figure S1), and (b) carbon isotope values in male lobsters were greater (+0.2‰ higher) in all tissue types compared to female lobsters (Figure S2a [supporting information]). We recorded high conditional *R* ^2^c (*R* ^2^c > 0.80) and low marginal *R* ^2^m values (*R* ^2^m = 0.01–0.15) for all δ^13^C models, and this indicates that considerable variation was contributed by random effects at the level of individual lobsters. Importantly, we detected a moderate to high correlation between antennae and abdomen tissues (Pearson's correlation = 0.778) and high correlation between leg and abdomen tissues (Pearson's correlation = 0.915). Therefore, with mathematical corrections applied, leg and antennae tissues will accurately estimate δ^13^C, which would otherwise be gained by sampling lobster abdomen tissues.

### 3.2 δ^15^N difference in lobster tissues

AICc model comparisons showed the best model consisting the null model (H0), and three models were also within ±2 AICc, which contained the following predictors: month collected, lobster tissue type and month + tissue type (Table 1B). It was notable that we observed consistent variation between months, meaning that there was a discernible pattern that δ^15^N varied between months (Figure 1B; Table 1B). Leg tissues showed marginally higher δ^15^N (+0.1‰) values than antennae tissues, which were closer to abdomen tissues (Figures S1 and S2 [supporting information]). We did not calculate the correction factors for δ^15^N appendage tissues because the null model was selected, and there were only marginal differences in isotope values based on lobster tissue (Figure 1B; Table 1B). Although we did not record any important effect of lobster size or sex, we did observe two trends: (a) δ^15^N decreased with increasing lobster size in all tissues (Figure S1), and (b) nitrogen isotope values in male lobsters increased (+0.2‰ higher) in all tissue types compared to female lobsters (Figure S2b [supporting information]). We recorded low conditional (*R* ^2^c < 0.28) and marginal (*R* ^2^m < 0.15) *R* ^2^ values for all δ^15^N models, and this indicates that the random and fixed effects contributed little variation at the level of individual lobsters. Importantly, we detected little to no correlation between antennae and abdomen (Pearson's correlation = 0.03) and leg and abdomen tissues (Pearson's correlation <0.01). However, because the overall difference between the antennae abdomen tissues was negligible, this suggests that any tissue can be sampled and, therefore, that leg and antennae tissues will not misrepresent δ^15^N, which would otherwise be gained by sampling lobster abdomen tissues.

## 4 DISCUSSION

We found that after mathematical correction for variation among leg and abdominal tissue, stable isotope analysis of non‐lethally excised limb tissue yields reliable estimates of abdominal isotope ratios. Correction values for between‐tissue variation and tissue characteristics are regularly used in isotope studies (e.g., lipid correction^32^, ^40^, ^41^, ^42^). For instance, mathematical corrections to standardise isotopic ratios have been used previously for different tissues of *Panulirus argus* ^18^ and *Homarus americanus* (Table 2).^31^ Our model‐predicted estimates show that corrections of −0.3 ± 0.2‰ SE (antennae) and −0.1 ± 0.2‰ SE (leg) for δ^13^C can standardise isotopic ratios from non‐lethally obtained appendage tissues of *S. verreauxi*. These estimates for *S. verreauxi* will be more reliable than previous work with *P. argus*, which had a sample size of n = 10,^18^ as we collected a much larger data set of lobster abdomen, antennae and leg tissues (n = 76) (Table 2). For δ^15^N, corrections are unnecessary given that we found negligible difference between tissue types.

**TABLE 2:** Isotope values for (A) δ^13^C and (B) δ^15^N appendage tissues from the lobsters *Panulirus argus* (Caribbean spiny lobster), *Panulirus cygnus* (western spiny lobster), the crab *Callinectes sapidus* (Chesapeake blue crab) and the crayfish *Cherax destructor* (common yabby) across different body tissues, taken from the literature for comparison with values from stable isotope analysis of *Sagmariasus verreauxi* (eastern rock lobster)

A								
Isotope	Species	Sizes (mm)	Tissue	Range (δ^13^C‰)	Mean (δ^13^C‰)	SE	n	Literature source
^13^C	*S. verreauxi*	104–137	Abdomen	−17.4 to −13.0	−15.1	0.1	76	This study
–	*S. verreauxi*	104–137	Antennae	−17.2 to −12.4	−14.8	0.1	76	This study
–	*S. verreauxi*	104–137	Leg	−16.9 to −12.7	−15.0	0.1	76	This study
–	*J* *asus* *paulensis*	65–105	Antennae	−18.2 to −17.0	−17.9	0.4	40	^10^
–	*Jasus edwardsii*	80–>120	Leg	nd	−15.5	0.2	30	^11^
–	*P. argus*	95–107	Abdomen	nd	−6.3	0.4	10	^18^
–	*P. argus*	95–107	Antennae	nd	−7.8	0.3	9	^18^
–	*P. argus*	95–107	Leg	nd	−7.0	0.3	10	^18^
–	*P. cygnus*	53–145	Tail	−19.2 to −18.1	−18.5	0.3	16	^19^
–	*P. cygnus*	53–145	Leg	−19.5 to −18.3	−18.9	0.2	16	^19^
–	*C. sapidus*	nd	Abdomen	−16.0 to −15.1	−16	0.1	11	^14^
–	*C. sapidus*	nd	Claw	9.8	−21.3	0.7	35	^30^
–	*C. sapidus*	nd	Leg	6.6	−23.8	0.9	35	^30^
–	*C. destructor*	nd	Carapace	nd	2.9	0.4	108	^21^
–	*C. destructor*	nd	Claw	nd	1.4	0.4	108	^21^

B								
Isotope	Species	Sizes (mm)	Tissue	Range (δ^15^N‰)	Mean (δ^15^N‰)	SE	n	Literature source
^15^N	*S. verreauxi*	104–137	Abdomen	10.6–13.6	11.9	0.1	76	This study
*–*	*S. verreauxi*	104–137	Antennae	10.4–13.3	11.9	0.1	76	This study
*–*	*S. verreauxi*	104–137	Leg	10.6–13.8	12.11	0.1	76	This study
*–*	*J. paulensis*	65–105	Antennae	10.2–12.9	11.2	1.0	40	^10^
*–*	*J. edwardsii*	80–>120	Leg	nd	13.5	0.2	30	^11^
*–*	*P. argus*	95–107	Abdomen	nd	6	0.2	10	^18^
*–*	*P. argus*	95–107	Antennae	nd	5.7	0.2	9	^18^
*–*	*P. argus*	95–107	Leg	nd	7.8	0.2	10	^18^
*–*	*P. cygnus*	53–145	Tail	10.3–12.1	10.9	0.2	16	^19^
*–*	*P. cygnus*	53–145	Leg	10.6–12.3	11.5	0.2	16	^19^
*–*	*C. sapidus*	nd	Abdomen	10.7–11.4	11.2	0.1	10	^14^
*–*	*C. sapidus*	nd	Claw	10.3–14.8	12.5	0.7	35	^30^
*–*	*C. sapidus*	nd	Leg	9.1–13.8	11.4	0.3	35	^30^
*–*	*C. destructor*	nd	Carapace	nd	−1	0.3	108	^21^
*–*	*C. destructor*	nd	Claw	nd	2.5	0.4	108	^21^

*Note*: Body size of species used in comparisons is shown with the range of isotopic values and means ± standard error of the mean.

Abbreviation: nd, no data.

When interpreting diets using isotopic Bayesian analysis, diet‐tissue offsets of 0‰–1.0‰ for δ^13^C^19^ and 3.0‰–4.0‰ for δ^15^N^11^, ^12^ are typically employed. Importantly, our results show that variances in δ^13^C and δ^15^N among the leg, antennae and abdomen tissues of lobsters are within the range of uncertainty typically used in estimating predator diets via Bayesian modelling.^11^, ^12^, ^19^ Given our results show that using leg or antennae appendage tissue will not misrepresent lobster food source or trophic level, this confirms the viability of non‐lethal sampling *S. verreauxi* diets via limb removal. Notably, abdomen tissue samples had lower δ^13^C and δ^15^N values than other tissues, and this is similarly reflected in past work with other various crustacea, including crabs, lobsters and prawns (Table 2).^14^, ^17^, ^18^, ^19^ Tissues inside the carapace are maintained long term compared to appendages which are frequently lost and regrown in the wild.^26^, ^27^ It has been suggested that well‐protected tissues found inside the thorax of lobsters, which persist throughout a lobster’s lifetime, have a different rate of isotopic turnover than more vulnerable tissues like limbs^31^, ^43^ which will likely be lost at some stage while escaping predation,^26^, ^27^ and this may partially explain a trend observed in our data. We recorded stable isotope ratios that were identical between different tissues in 24 lobsters (31.6%) for δ^13^C and 10 lobsters (13.2%) for δ^15^N. Another 10 lobsters had similar but non‐identical carbon and nitrogen isotope values for all tissues (abdomen, leg and antennae). This aligns with work on other crustaceans, which suggests that regrown limbs synthesise proteins differently to long‐surviving tissues.^28^, ^29^ This could explain the trend of identical isotope values between and within appendage and abdomen tissues, which we observed in some lobsters. However, explicitly testing this was outside the scope of this study, and investigating isotopic turnover rates of the different lobster tissues is an area for future work.

Non‐lethal sampling through limb removal will be useful especially for *S. verreauxi* because the species has shown 100% survivorship where limb loss occurred^27^ and discard mortality was less than 2% in trapping studies.^26^ Therefore, the removal of a single walking leg from *S. verreauxi* *in situ* will be a low‐impact sampling design. Similar to other studies,^18^, ^19^ we suggest that taking a leg versus an antenna will have the least possible impact on lobster livelihoods because antennae (of which there are two) are used in sensing and defence, whereas walking legs (of which there are eight) are used primarily for locomotion.^3^ Further, because lobsters can defend themselves from predators just as effectively after losing an appendage,^44^ we are confident that limb removal will not impact lobster populations. However, it should be noted that for *S. verreauxi*, limbs regrow on the next moult at a cost to overall CL and the length of the regrown limb, which can be diminished greatly.^27^, ^45^ Finally, the application of non‐lethal sampling provides avenues for the contribution of citizen science to understanding lobster food webs and will allow researchers to undertake fieldwork in ecologically sensitive areas such as marine reserves.

## PERMITS

Animals were collected using NSW DPI Scientific Collection Permit P13/0037‐2.0.

### PEER REVIEW

The peer review history for this article is available at https://publons.com/publon/10.1002/rcm.9435.

## Supporting information


**FIGURE S1** Relationship between stable isotope values and lobster size (carapace length [CL], mm) for (A–C) ^13^C and (D–F) ^15^N in three different body tissues taken from 76 *Sagmariasus verreauxi* (eastern rock lobster). Values for the “abdomen,” “antennae” and “leg” tissues are shown on the *y*‐axis. Month collected is shown by different shapes, and sex is shown by filled (female ♀) and unfilled (male ♂) shapes. The solid line represents the predicted likelihood of obtaining isotope values at different lobster sizes and shows no significant effect. The grey shaded area indicates standard error margins of the predicted curve
**FIGURE S2** GLMM‐predicted values for (A) δ^13^C and (b) δ^15^N shown by tissue type. Sex is represented by black (female) and grey (males). Values are mean ± standard error. Tissue type was either in the best model or in models within ±2 AICc of the best model for both isotopes, whereas sex was not
**TABLE S1** Raw isotope data for leg tissues of 76 *Sagmariasus verreauxi* (eastern rock lobster) collected in Shellharbour, New South Wales, in 2020 from May to September. Tissue type (tissue), individual lobsters (ID) and δ^13^C and δ^15^N are shown. Values for the different tissue types (leg, antennae and abdomen) are partitioned

## Data Availability

The data that supports the findings of this study are available in the supplementary material of this article.
