# Supplementary material for: Non‐lethal sampling does not misrepresent trophic level or dietary sources for Sagmariasus verreauxi (eastern rock lobster)
Source: Rapid Commun Mass Spectrom. 2022 Dec 18;37(4):e9435. doi: 10.1002/rcm.9435 (PMC10078346; doi:10.1002/rcm.9435)
Supplement: Supplementary file 1 — FIGURE S1 Relationship between stable isotope values and lobster size (carapace length [CL], mm) for (A–C) 13C and (D–F) 15N in three different body tissues taken from 76 Sagmariasus verreauxi (eastern rock lobster). Values for the “abdomen,” “antennae” and “leg” tissues are shown on the y‐axis. Month collected is shown by different shapes, and sex is shown by filled (female ♀) and unfilled (male ♂) shapes. The solid line represents the predicted likelihood of obtaining isotope values at different lobster sizes and shows no significant effect. The grey shaded area indicates standard error margins of the predicted curve FIGURE S2 GLMM‐predicted values for (A) δ13C and (b) δ15N shown by tissue type. Sex is represented by black (female) and grey (males). Values are mean ± standard error. Tissue type was either in the best model or in models within ±2 AICc of the best model for both isotopes, whereas sex was not TABLE S1 Raw isotope data for leg tissues of 76 Sagmariasus verreauxi (eastern rock lobster) collected in Shellharbour, New South Wales, in 2020 from May to September. Tissue type (tissue), individual lobsters (ID) and δ13C and δ15N are shown. Values for the different tissue types (leg, antennae and abdomen) are partitioned [file RCM-37-0-s001.docx]

**
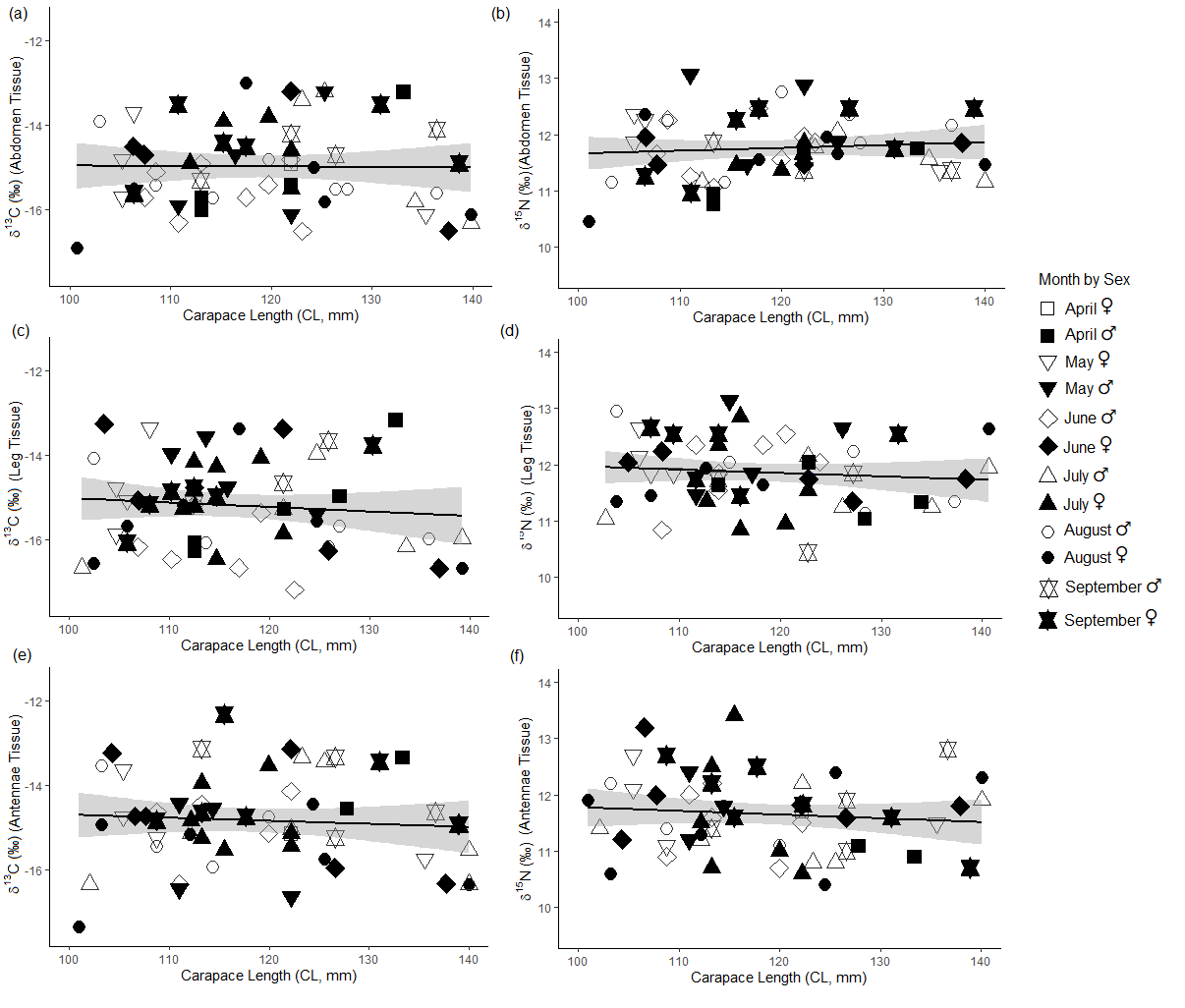
**

**Fig. S1.** Relationship between stable isotope values and lobster size (Carapace Length, CL, mm) for (a) to (c) ^13^C and (d) to (f) ^15^N in three different body tissues taken from 76 *Sagmariasus verreauxi* (eastern rock lobster). Values for the tissues ‘abdomen’, ‘antennae’ and ‘leg’ are shown on the y-axis. Month collected is shown by different shapes and sex is shown by filled (female ♀) and un-filled (male ♂) shapes. The solid line represents the predicted likelihood of obtaining isotope values at different lobster size and shows no significant effect. The grey shaded area indicates standard error margins of the predicted curve.

**
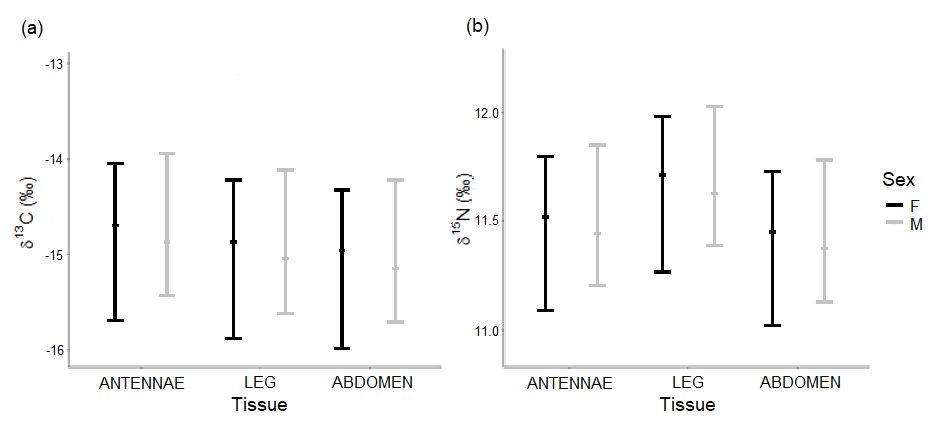
Fig. S2.** GLMM predicted values for (a) δ^13^C and (b) δ^15^N shown by tissue type. Sex is represented by black (female) and grey (males). Values are mean ± standard error. Tissue type was either in the best model or in models within ±2 AICc of the best model for both isotopes while sex was not.

**Table S1.** Raw isotope data for leg tissues of 76 *Sagmariasus verreauxi* (eastern rock lobster) collected in Shellharbour, NSW in 2020 from May to September. Tissue type (Tissue), individual lobsters (ID) and δ^13^C and δ^15^N are shown. Values for the different tissue types (leg, antennae, abdomen) are partitioned.

| **Tissue** | **ID** | δ**^13^C**‰ | δ**^15^N**‰ |
| --- | --- | --- | --- |
| LEG | A10-LEG | -14.9 | 13.1 |
| LEG | A11-LEG | -14.1 | 11.5 |
| LEG | A12-LEG | -14.4 | 11.8 |
| LEG | A13-LEG | -14.5 | 12.6 |
| LEG | A15-LEG | -14.7 | 11.6 |
| LEG | A16-LEG | -14.9 | 11.9 |
| LEG | A55-LEG | -14.8 | 12.4 |
| LEG | A56-LEG | -15.3 | 11.5 |
| LEG | A57-LEG | -15.9 | 13.2 |
| LEG | A58-LEG | -16.1 | 11.5 |
| LEG | A59-LEG | -14.6 | 12.7 |
| LEG | A60-LEG | -14.5 | 12.8 |
| LEG | A61-LEG | -16.1 | 13.0 |
| LEG | A62-LEG | -15.6 | 12.3 |
| LEG | A63-LEG | -14.6 | 12.2 |
| LEG | A64-LEG | -14.5 | 11.9 |
| LEG | A65-LEG | -14.5 | 11.6 |
| LEG | A67-LEG | -14.8 | 12.1 |
| LEG | A68-LEG | -13.1 | 11.9 |
| LEG | A69-LEG | -13.3 | 11.6 |
| LEG | A70-LEG | -16.1 | 11.6 |
| LEG | A72-LEG | -14.5 | 12.1 |
| LEG | A95-LEG | -16.4 | 11.9 |
| LEG | B06-LEG | -15.0 | 11.0 |
| LEG | B07-LEG | -15.2 | 12.2 |
| LEG | B08-LEG | -15.4 | 11.7 |
| LEG | B09-LEG | -15.3 | 11.2 |
| LEG | B10-LEG | -15.0 | 11.6 |
| LEG | B11-LEG | -12.9 | 11.5 |
| LEG | B12-LEG | -14.9 | 11.7 |
| LEG | B13-LEG | -14.6 | 11.5 |
| LEG | B15-LEG | -13.7 | 11.4 |
| LEG | B16-LEG | -14.6 | 12.0 |
| LEG | B17-LEG | -15.6 | 11.7 |
| LEG | B19-LEG | -14.9 | 11.9 |
| LEG | B23-LEG | -14.7 | 12.6 |
| LEG | B24-LEG | -15.7 | 12.6 |
| LEG | B25-LEG | -13.4 | 12.3 |
| LEG | B28-LEG | -16.0 | 11.6 |
| LEG | B31-LEG | -16.0 | 12.0 |
| LEG | B32-LEG | -15.1 | 12.7 |
| LEG | B33-LEG | -16.9 | 10.6 |
| LEG | B38-LEG | -16.3 | 11.3 |
| LEG | B40-LEG | -16.4 | 11.8 |
| LEG | B42-LEG | -13.8 | 11.1 |
| LEG | B44-LEG | -14.9 | 11.3 |
| LEG | B26-LEG | -16.4 | 12.8 |
| LEG | B27-LEG | -15.3 | 12.5 |
| LEG | B22-LEG | -15.1 | 12.6 |
| LEG | B30-LEG | -15.1 | 12.4 |
| LEG | B29-LEG | -14.8 | 12.4 |
| LEG | B39-LEG | -16.0 | 12.2 |
| LEG | B43-LEG | -15.6 | 13.8 |
| LEG | B34-LEG | -15.4 | 12.4 |
| LEG | A06-LEG | -15.3 | 12.8 |
| LEG | A05-LEG | -15.5 | 12.3 |
| LEG | B41-LEG | -15.3 | 12.9 |
| LEG | B21-LEG | -13.4 | 11.9 |
| LEG | A04-LEG | -15.8 | 12.2 |
| LEG | A14-LEG | -14.4 | 11.7 |
| LEG | B20-LEG | -15.6 | 12.3 |
| LEG | B37-LEG | -14.7 | 12.6 |
| LEG | B36-LEG | -13.5 | 12.7 |
| LEG | B18-LEG | -13.9 | 12.0 |
| LEG | B14-LEG | -14.0 | 11.1 |
| LEG | B35-LEG | -14.9 | 12.6 |
| LEG | A71-LEG | -14.5 | 12.0 |
| LEG | A66-LEG | -16.0 | 12.4 |
| LEG | A73-LEG | -14.5 | 12.6 |
| LEG | A02-LEG | -15.0 | 12.1 |
| LEG | A96-LEG | -16.2 | 12.5 |
| LEG | A01-LEG | -15.8 | 12.2 |
| LEG | A07-LEG | -15.2 | 11.7 |
| LEG | A03-LEG | -14.8 | 12.9 |
| LEG | A08-LEG | -13.8 | 13.1 |
| LEG | A09-LEG | -12.7 | 12.3 |
| ANTENNAE | A10-ANT | -14.9 | 12.7 |
| ANTENNAE | A11-ANT | -13.6 | 10.4 |
| ANTENNAE | A12-ANT | -13.5 | 11.1 |
| ANTENNAE | A13-ANT | -12.7 | 11.4 |
| ANTENNAE | A15-ANT | -12.4 | 12.0 |
| ANTENNAE | A16-ANT | -13.2 | 11.8 |
| ANTENNAE | A55-ANT | -13.2 | 12.0 |
| ANTENNAE | A56-ANT | -13.1 | 12.0 |
| ANTENNAE | A57-ANT | -13.7 | 12.7 |
| ANTENNAE | A58-ANT | -15.3 | 12.0 |
| ANTENNAE | A59-ANT | -13.7 | 12.4 |
| ANTENNAE | A60-ANT | -13.7 | 13.1 |
| ANTENNAE | A61-ANT | -15.7 | 12.6 |
| ANTENNAE | A62-ANT | -14.8 | 12.5 |
| ANTENNAE | A63-ANT | -13.3 | 13.3 |
| ANTENNAE | A64-ANT | -14.7 | 11.6 |
| ANTENNAE | A65-ANT | -13.7 | 11.6 |
| ANTENNAE | A67-ANT | -14.2 | 11.7 |
| ANTENNAE | A68-ANT | -13.2 | 12.2 |
| ANTENNAE | A69-ANT | -13.0 | 12.2 |
| ANTENNAE | A70-ANT | -15.7 | 12.6 |
| ANTENNAE | A72-ANT | -14.8 | 12.3 |
| ANTENNAE | A95-ANT | -16.4 | 12.2 |
| ANTENNAE | B06-ANT | -16.3 | 11.5 |
| ANTENNAE | B07-ANT | -15.5 | 12.5 |
| ANTENNAE | B08-ANT | -16.0 | 10.9 |
| ANTENNAE | B09-ANT | -15.7 | 11.1 |
| ANTENNAE | B10-ANT | -15.4 | 11.8 |
| ANTENNAE | B11-ANT | -13.4 | 11.3 |
| ANTENNAE | B12-ANT | -15.2 | 10.9 |
| ANTENNAE | B13-ANT | -14.7 | 11.2 |
| ANTENNAE | B15-ANT | -13.5 | 11.2 |
| ANTENNAE | B16-ANT | -15.1 | 11.0 |
| ANTENNAE | B17-ANT | -15.5 | 11.0 |
| ANTENNAE | B19-ANT | -15.0 | 11.5 |
| ANTENNAE | B23-ANT | -14.5 | 12.7 |
| ANTENNAE | B24-ANT | -15.6 | 11.4 |
| ANTENNAE | B25-ANT | -13.4 | 12.0 |
| ANTENNAE | B28-ANT | -16.5 | 12.0 |
| ANTENNAE | B31-ANT | -16.0 | 11.5 |
| ANTENNAE | B32-ANT | -15.2 | 11.1 |
| ANTENNAE | B33-ANT | -17.2 | 10.7 |
| ANTENNAE | B38-ANT | -16.1 | 11.5 |
| ANTENNAE | B40-ANT | -16.4 | 11.2 |
| ANTENNAE | B42-ANT | -13.6 | 11.4 |
| ANTENNAE | B44-ANT | -14.8 | 10.9 |
| ANTENNAE | B26-ANT | -16.4 | 12.7 |
| ANTENNAE | B27-ANT | -15.5 | 12.5 |
| ANTENNAE | B22-ANT | -15.0 | 12.3 |
| ANTENNAE | B30-ANT | -15.1 | 12.2 |
| ANTENNAE | B29-ANT | -14.8 | 12.4 |
| ANTENNAE | B39-ANT | -15.8 | 11.7 |
| ANTENNAE | B43-ANT | -16.2 | 13.0 |
| ANTENNAE | B34-ANT | -15.7 | 12.2 |
| ANTENNAE | A06-ANT | -15.8 | 12.8 |
| ANTENNAE | A05-ANT | -15.6 | 12.3 |
| ANTENNAE | B41-ANT | -15.0 | 12.5 |
| ANTENNAE | B21-ANT | -13.3 | 12.0 |
| ANTENNAE | A04-ANT | -16.0 | 12.2 |
| ANTENNAE | A14-ANT | -14.2 | 11.5 |
| ANTENNAE | B20-ANT | -15.7 | 12.1 |
| ANTENNAE | B37-ANT | -15.0 | 12.6 |
| ANTENNAE | B36-ANT | -13.5 | 12.0 |
| ANTENNAE | B18-ANT | -13.9 | 11.6 |
| ANTENNAE | B14-ANT | -13.9 | 11.8 |
| ANTENNAE | B35-ANT | -15.1 | 11.9 |
| ANTENNAE | A71-ANT | -14.5 | 12.0 |
| ANTENNAE | A6-ANT | -15.7 | 11.8 |
| ANTENNAE | A73-ANT | -14.9 | 11.7 |
| ANTENNAE | A02-ANT | -15.0 | 12.1 |
| ANTENNAE | A96-ANT | -16.4 | 12.4 |
| ANTENNAE | A01-ANT | -15.5 | 12.0 |
| ANTENNAE | A07-ANT | -14.9 | 12.1 |
| ANTENNAE | A03-ANT | -14.8 | 12.5 |
| ANTENNAE | A08-ANT | -13.6 | 12.6 |
| ANTENNAE | A09-ANT | -13.0 | 11.7 |
| ABDOMEN | LOB-A11 | -14.7 | 13.2 |
| ABDOMEN | LOB-B15 | -13.2 | 12.2 |
| ABDOMEN | LOB-A55 | -15.1 | 12.8 |
| ABDOMEN | LOB-A69 | -13.0 | 11.4 |
| ABDOMEN | LOB-A72 | -14.8 | 13.6 |
| ABDOMEN | LOB-B11 | -13.2 | 11.9 |
| ABDOMEN | LOB-B24 | -15.8 | 12.8 |
| ABDOMEN | LOB-B31 | -15.7 | 11.9 |
| ABDOMEN | LOB-B38 | -16.4 | 12.3 |
| ABDOMEN | LOB-A95 | -16.5 | 12.0 |
| ABDOMEN | LOB-B28 | -16.9 | 12.2 |
| ABDOMEN | LOB-B40 | -15.9 | 11.7 |
| ABDOMEN | LOB-A13 | -14.8 | 12.9 |
| ABDOMEN | LOB-A10 | -13.3 | 12.2 |
| ABDOMEN | LOB-B17 | -15.5 | 11.8 |
| ABDOMEN | LOB-B10 | -15.0 | 12.2 |
| ABDOMEN | LOB-B25 | -13.5 | 11.4 |
| ABDOMEN | LOB-B33 | -17.4 | 12.3 |
| ABDOMEN | LOB-B32 | -15.4 | 11.7 |
| ABDOMEN | LOB-B08 | -16.0 | 11.9 |
| ABDOMEN | LOB-A63 | -14.1 | 13.0 |
| ABDOMEN | LOB-A61 | -16.7 | 12.2 |
| ABDOMEN | LOB-A60 | -14.8 | 12.5 |
| ABDOMEN | LOB-A12 | -14.6 | 11.9 |
| ABDOMEN | LOB-B23 | -14.7 | 12.8 |
| ABDOMEN | LOB-B12 | -15.2 | 12.1 |
| ABDOMEN | LOB-B42 | -13.8 | 11.5 |
| ABDOMEN | LOB-B09 | -15.8 | 11.8 |
| ABDOMEN | LOB-A65 | -14.0 | 12.1 |
| ABDOMEN | LOB-A57 | -16.5 | 12.8 |
| ABDOMEN | LOB-A68 | -13.2 | 11.6 |
| ABDOMEN | LOB-A59 | -14.8 | 12.0 |
| ABDOMEN | LOB-B19 | -14.9 | 11.5 |
| ABDOMEN | LOB-A58 | -15.8 | 11.9 |
| ABDOMEN | LOB-A15 | -14.4 | 12.4 |
| ABDOMEN | LOB-B07 | -15.9 | 12.4 |
| ABDOMEN | LOB-B06 | -15.6 | 12.6 |
| ABDOMEN | LOB-B16 | -15.2 | 12.2 |
| ABDOMEN | LOB-A62 | -15.7 | 12.0 |
| ABDOMEN | LOB-A70 | -16.4 | 12.5 |
| ABDOMEN | LOB-A56 | -15.3 | 12.5 |
| ABDOMEN | LOB-A67 | -14.2 | 11.9 |
| ABDOMEN | LOB-A16 | -15.3 | 12.0 |
| ABDOMEN | LOB-A64 | -14.5 | 12.0 |
| ABDOMEN | LOB-B13 | -14.7 | 11.6 |
| ABDOMEN | LOB-B44 | -15.2 | 11.6 |
| ABDOMEN | B27-CP | -15.4 | 11.6 |
| ABDOMEN | B22-CP | -15.3 | 11.8 |
| ABDOMEN | B30-CP | -14.7 | 11.3 |
| ABDOMEN | B29-CP | -14.7 | 11.6 |
| ABDOMEN | B39-CP | -15.9 | 11.4 |
| ABDOMEN | B43-CP | -16.1 | 12.5 |
| ABDOMEN | B34-CP | -15.5 | 11.8 |
| ABDOMEN | A06-CP | -15.8 | 11.8 |
| ABDOMEN | A05-CP | -15.7 | 11.5 |
| ABDOMEN | B41-CP | -15.6 | 11.3 |
| ABDOMEN | B21-CP | -13.4 | 11.2 |
| ABDOMEN | A04-CP | -15.7 | 11.3 |
| ABDOMEN | A14-CP | -14.4 | 10.6 |
| ABDOMEN | B20-CP | -16.0 | 11.3 |
| ABDOMEN | B37-CP | -15.3 | 11.4 |
| ABDOMEN | B36-CP | -13.5 | 11.9 |
| ABDOMEN | B18-CP | -14.0 | 11.0 |
| ABDOMEN | B14-CP | -14.3 | 10.7 |
| ABDOMEN | B35-CP | -15.0 | 11.1 |
| ABDOMEN | A71-CP | -14.9 | 11.2 |
| ABDOMEN | A66-CP | -15.9 | 11.0 |
| ABDOMEN | A73-CP | -15.2 | 10.8 |
| ABDOMEN | A02-CP | -14.5 | 10.8 |
| ABDOMEN | A96-CP | -16.3 | 11.4 |
| ABDOMEN | A01-CP | -15.4 | 11.3 |
| ABDOMEN | A07-CP | -14.6 | 11.0 |
| ABDOMEN | A03-CP | -14.8 | 11.5 |
| ABDOMEN | A08-CP | -13.9 | 11.3 |
| ABDOMEN | A09-CP | -13.1 | 11.8 |
